# Unmasking the Uncommon: A Case Report of Scrotal Epidermoid Cysts in a Nine-Year-Old Boy

**DOI:** 10.7759/cureus.41045

**Published:** 2023-06-27

**Authors:** Arisha Ahmed, Vasudeo Ridhorkar, Deepak Goel, Ashay Suryawanshi

**Affiliations:** 1 Surgery, KIMS-Kingsway Hospitals, Nagpur, IND; 2 Urology, KIMS-Kingsway Hospitals, Nagpur, IND; 3 Pediatric Surgery, KIMS-Kingsway Hospitals, Nagpur, IND

**Keywords:** urology surgery, paediatric surgery, pelvic mass, extra-testicular tumor, scrotal mass, epidermoid cysts

## Abstract

Scrotal epidermoid cysts are rare. Intratesticular epidermoid cysts are more common than extra scrotal cysts and are the most commonest benign tumors of the testicles. Midline scrotal raphe cysts are reported, but only a few have intrapelvic extensions deep into the pelvis. A nine-year-old boy presented with a painless scrotal swelling. Magnetic resonance imaging (MRI) of the pelvis confirmed the cystic nature with an extension of the swelling up to the base of the prostate. On surgical exploration, the cyst had a tapering stalk with cranial extension up to the base of the prostate. The patient underwent an excision of the cyst and made an uneventful recovery and was asymptomatic at the end of three months of surgery. The histopathology of the lesion was typical of an epidermoid cyst. Extratesticular scrotal epidermoid cysts with pelvic extension are a rarity with less than five cases reported in the literature. Our case stands to be the youngest reported case of a scrotal epidermoid cyst based on our knowledge. Scrotal epidermoid cysts are a very rare and benign entity, and upon recognition and confirmation of the extent of spread, extratesticular scrotal cysts can safely be removed. No other management may be required with no recurrences reported.

## Introduction

Scrotal epidermoid cysts are rare. Epidermoid cysts are lined with squamous epithelium and contain proteinaceous debris of desquamated epithelium. Intratesticular epidermoid cysts are more common than extratesticular scrotal cysts. Such cysts are the commonest benign tumors of the testicles. Midline scrotal raphe cysts are reported, but only a few have intrapelvic extensions deep into the pelvis.

## Case presentation

A nine-year-old boy presented with a scrotal swelling detected by the parents. The lump was noticed about a week before his presentation to the hospital. It was painless, and no rapid increase in size was noted.

**Figure 1:**
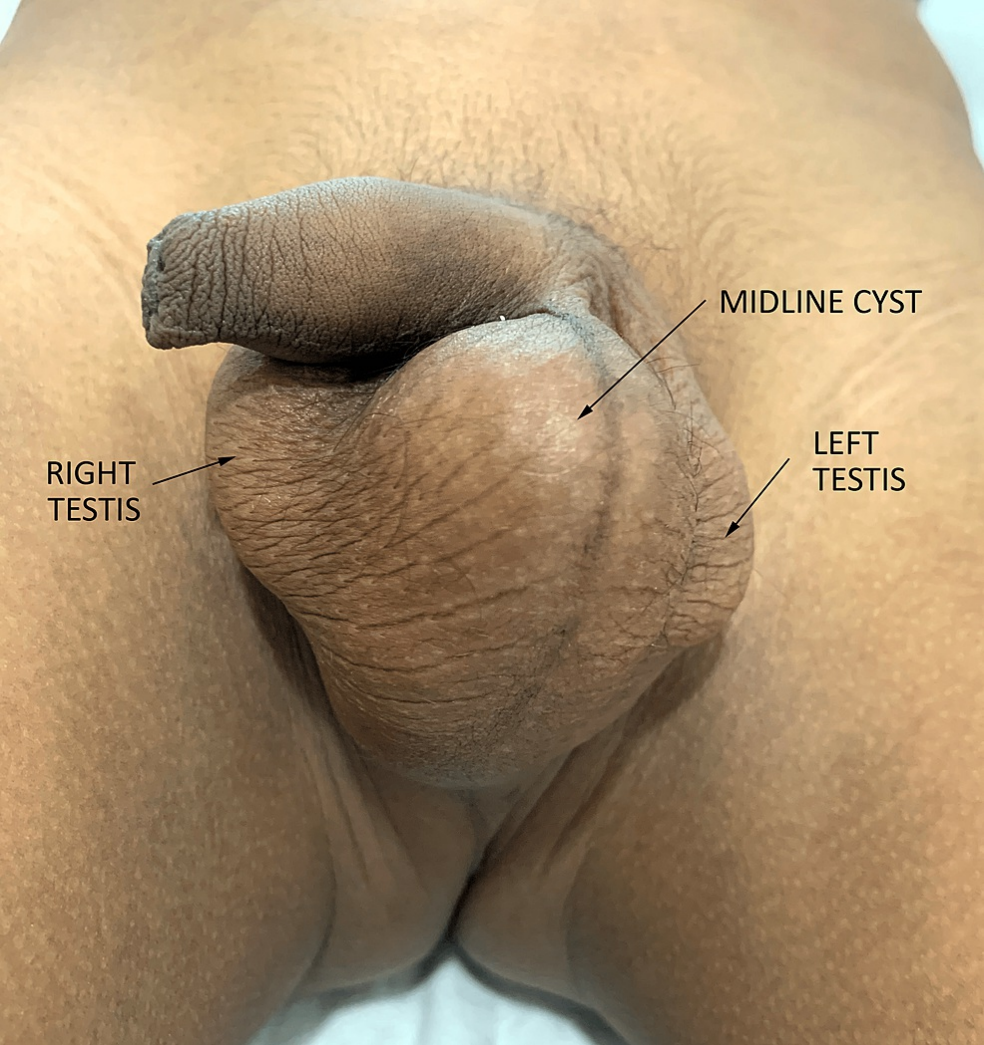
Gross examination findings with labels.

**Figure 2:**
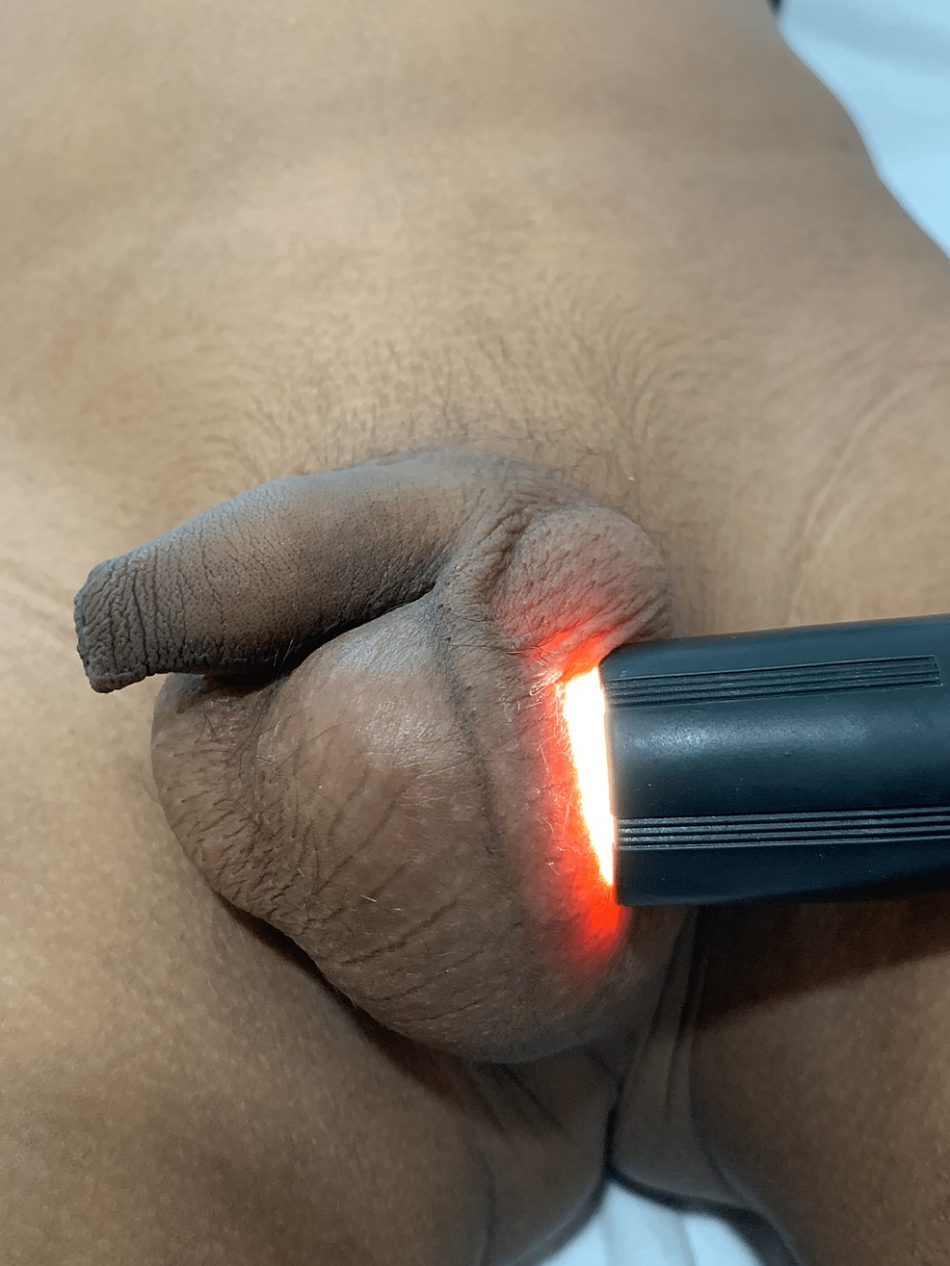
Transillumination test.

On examination, the swelling was found to be nontender, discrete, cystic in consistency, located in the midline, extending to the right side, and separate from the right testicle (Figure 1). It was mobile and transillumination was absent, as shown in Figure 2.

Ultrasound of the scrotum showed a solitary, thin-walled, unilocular isoechoic mass in the scrotum. It was observed to be distinct and separate from both testicles.

**Figure 3:**
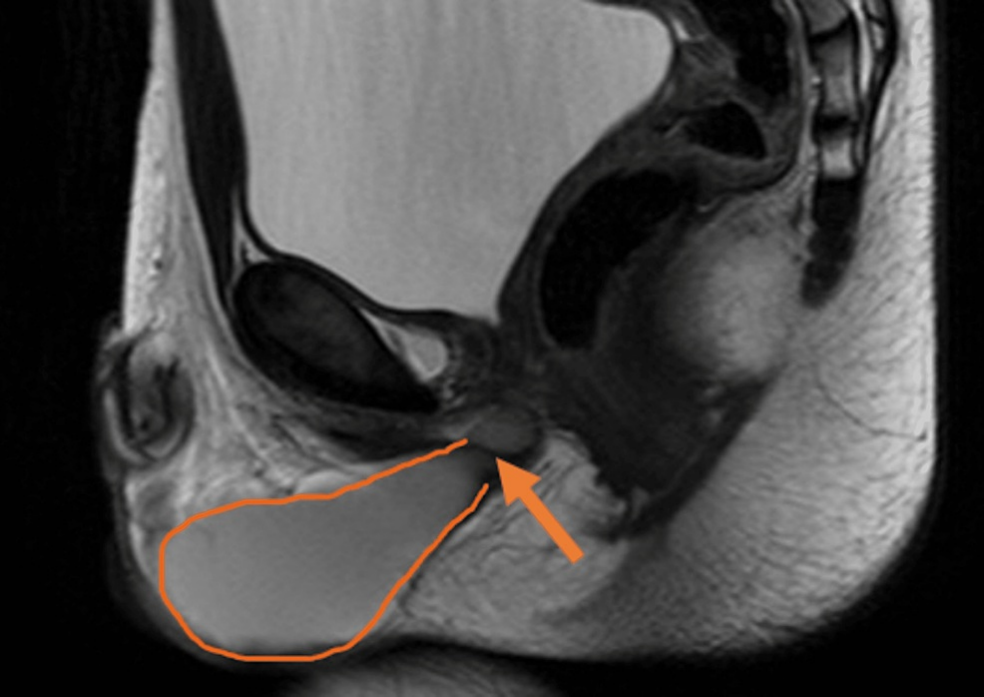
Pelvic MRI. The boundaries of the cyst have been outlined in orange, and the stalk connecting it to the base of the prostate has been marked with an orange arrow. MRI, magnetic resonance imaging

On pelvic MRI, the lesion was cystic in nature and showed a stalk-like extension extending superiorly up to the base of the prostate, traversing the pelvic floor. The fat planes were preserved. It exhibited homogeneous T1, T2, and STIR hyperintensity on the MRI images, with no suppression of signal observed on T1 FS images. This was suggestive of a proteinaceous content. No lymphadenopathy or other abnormalities were noted on the MRI (Figure 3). The orange arrow shows the attachment of the cyst to the base of the prostate, and the orange outline shows the full cyst. 

The laboratory investigations were within normal limits. As the lesion was separate from the testicles, tumor markers were not sent.

**Figure 4:**
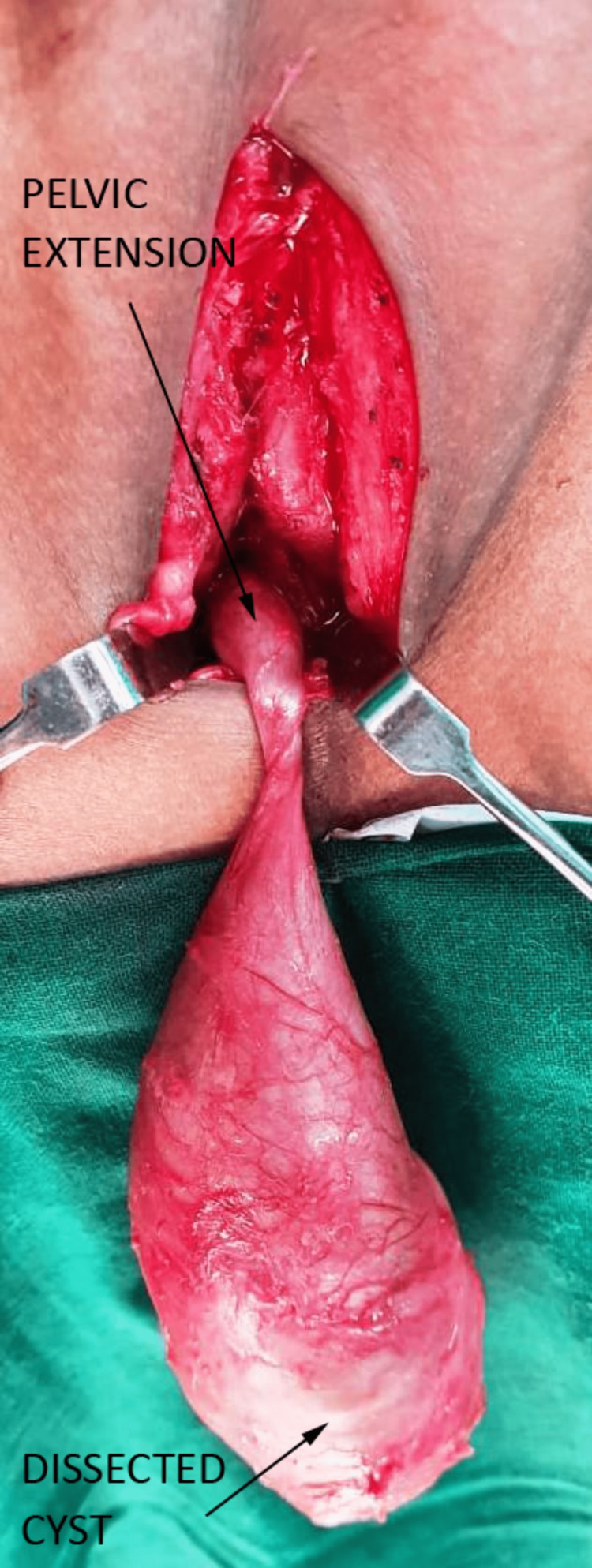
Intraoperative image with labels.

The patient underwent the procedure under general anesthesia. With the patient in a lithotomy position, a median raphe incision was taken. The cyst was exposed and dissected on all sides. The planes for dissection were easily separable, and there were no significant adhesions. The cyst had a tapering stalk going up beneath the urethra. The cranial extension was up to the base of the prostate, as shown in Figure 4. 

The cranial most fibrotic tip was disconnected, and the cyst was delivered out intact. There was no leakage or spillage from the cysts. The wound was closed in layers without a drain. The patient made an uneventful recovery.

**Figure 5:**
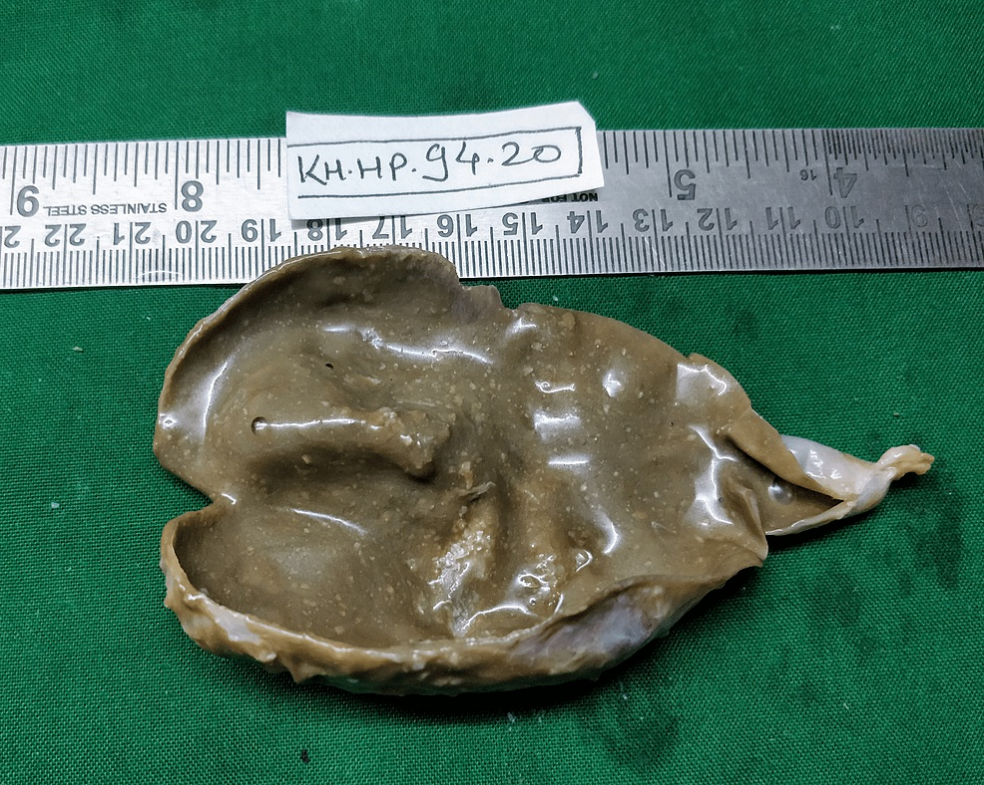
Gross cut section of the cyst.

The histopathology of the cyst was typical of an epidermoid cyst. Figure 5 shows the gross cut section of the cyst.

## Discussion

Epidermoid cysts are benign, well-circumscribed swelling, accounting for approximately 1% of all testicular tumors [1]. The intratesticular epidermoid cysts are more common than the extratesticular cysts. These tumors can occur at any age but are more common in the second to fourth decade of life [1]. They may be unilateral, bilateral, singular, or multiple and can be found at any place from the cranium to the anus in the midline [2,3]. The two main mechanisms by which the dermal epithelium reaches the deeper tissues and forms cysts are as follows: 

 1. Congenital: Here, the epithelium along the lines of epithelial fusion gets embedded during embryogenesis and later forms a cyst. These are usually midline and can be seen in the cranium to the anus along the lines of closure of the neural tube. Hence, such cysts are in the midline. The histogenesis is controversial, and sometimes, it is a monolayer teratoma or epithelial metaplasia.

 2. Acquired: Here, the epithelium is driven inside due to trauma or after surgical procedures.

Scrotal extratesticular cysts are uncommon and benign entities, and the nature of their presentation poses a challenge in diagnosis. Ultrasound is the primary investigation to explore scrotal content. Four patterns have been found in sonography: type I lesions have an onion-ring or whorled look, type II lesions are hyperechoic masses with posterior acoustic shadowing, type III lesions are hypoechoic masses with well-defined rims, and type IV lesions are heterogeneous lesions with ill-defined edges and a few sites of calcification. No reports of internal color Doppler signals have ever been made [1]. In our case, an ultrasound of the scrotum showed a solitary, thin-walled, unilocular iso-echoic mass, which was separate from both the testicles, resembling type III lesions.

The advancement of imaging modalities has enabled us to better appreciate the anatomy of such lesions. On MRI, such cysts are described as high-intensity, well-defined solid masses surrounded by a low-signal capsule on T2-weighted imaging [3]. Our finding on MRI showed a cystic lesion with a stalk extending up to the base of the prostate. 

Due to the nonneglectable radiation dose for scrotal lesions, computed tomography (CT) may raise the chance of developing radiation-induced secondary cancer in the future. Soft tissue lesions are better delineated on MRI. Therefore, it is preferable to use MRI instead of CT [2]. Pathologically, these lesions are squamous epithelium-lined cysts, containing keratin-like, cheesy white, flaky material, similar to those epidermoid cysts that occur intracutaneously [1,4].

One more such case has been reported by Van Gelderen et al., with a dermoid arising from the posterior aspect of the prostate and seminal vesicles [5]. In April 2013, Sağlam et al. published a similar case where the cyst was attached to the seminal vesicles [2]. This makes our case the third such case to be reported. This is the youngest patient with such a lesion to our knowledge. Our patient made an uneventful recovery and was asymptomatic at the end of three months of surgery.

## Conclusions

Scrotal epidermoid cysts are a very rare and benign entity. Upon recognition and confirmation of the extent of spread, extratesticular scrotal cysts can safely be removed. The nature of the presentation may pose a challenge to diagnosis. However, with the advancements in radiological techniques, it is now possible to better understand the extent and anatomy of the lesion, leading to more efficient surgical planning. In such cases, cyst excision is considered sufficient for management.
